# The effect of bovine vaccines against respiratory viruses administered either intranasal or intramuscular on broncho-alveolar fluid cells of heifers

**DOI:** 10.1080/01652176.2020.1870019

**Published:** 2021-01-18

**Authors:** Patricia S. Rossi, Rafael I. Mattei, Natali R. Schllemer, Gabriela R. Thomaz, Anna V. Antunes, Mauricio P. Virmond, Mari J. Taube, Heloisa G. Bertagnon

**Affiliations:** Departamento de Medicina Veterinária, Universidade Estadual do Centro Oeste (UNICENTRO), Guarapuava, PR, Brazil

**Keywords:** Bovine, heifer, vaccination, BALF, bronchoalveolar lavage, bovine respiratory complex (BRD)

## Abstract

**Background:**

The knowledge on bovine vaccines against respiratory viruses on bronchoalveolar fluid cells is scarce.

**Objective:**

To compare the effects of a commercial intranasal (IN) and intramuscular (IM) vaccine against bovine respiratory disease (BRD) complex viruses on bronchoalveolar fluid cells of healthy heifers.

**Methods:**

21 healthy heifers were assigned to three treatment groups: control (CO, N = 7), intranasally vaccinated (IN) (n = 7), and intramuscularly vaccinated (IM) (n = 7). The IN group received 1 mL of the commercial vaccine in each nostril once containing attenuated BoHV-1, bPIV-3, and BRSV. The IM group was vaccinated with two doses of 2 mL with an interval of 21 days of the commercial vaccine containing attenuated BoHV-1, bPIV-3, and BRSV plus inactivated BVDV. At day 0 (D0), before the first vaccine dose, and at D3, D7, and D21, after the last vaccine dose, airway bronchoscopy was performed to observe local irritation and collect bronchoalveolar lavage fluid (BALF). The bronchoalveolar count, cytological evaluation, bronchoalveolar cell oxidative metabolism, and total bronchoalveolar IgA and IgG were measured.

**Results:**

The IN vaccine increased neutrophil cellularity at D7 and D21 and total IgA at D3 in BALF. Total IgA in BALF also increased at D3 and oxidative metabolism of bronchoalveolar cells at D21 lowered compared to the CO group. Following IM vaccination there was no alteration of immunoglobulins or cell oxidative metabolism in BALF. Both vaccines reduced the number of alveolar macrophages.

**Conclusion:**

Both vaccines induced bronchoalveolar inflammation during the establishment of the vaccine immunity, which was more expressive in the IN protocol.

## 1. Introduction

Bovine respiratory disease (BRD) is a multifactorial disease related to a complex interaction between environmental stressors, immune susceptibility of animals, and respiratory pathogens, such as *bovine herpesvirus-1* (BoHV-1), *bovine parainfluenza virus type-3* (bPIV-3), *bovine respiratory syncytial virus* (BRSV), and *bovine viral diarrhea virus* (BVDV), associated with or without *Mannheimia haemolytica*, *Pasteurella multocida*, and *Haemophilus somnus* bacteria (Cusack et al. 2003; Edwards 2010).

Its high incidence has motivated the development of numerous preventive protocols, highlighting metaphylaxis (performed by a single dose of antibiotic before the stressful event) and or prophylaxis, in which animals receive one or two doses of a vaccine against respiratory pathogens 7 to 15 days before experiencing stressful events (Taylor et al. 2010).

As metaphylaxis can induce bacterial resistance and chemical residues in the animal, prophylaxis has become a more interesting measure for the control of BRD. Currently, most commercial vaccines are directed against viral agents BoHV-1, BVDV, bPIV-3, and BRSV, indicated for parenteral (Edwards 2010; Neutra and Kozlowski 2006) or intranasal (IN) administration (Ellis et al. 2007; Xue et al. 2010; Socha et al. 2013; Cortese et al. 2017).

IN vaccines are a potential alternative because they do not cause pain at the application site, can be administered in a single dose on the day of the stressful event, and the use of this application route shows an excellent ability to induce local IgA responses and protection against pathogens (Neutra and Kozlowski 2006; Osman et al. 2018). However, Dou et al. (2015) suggested that this pathway mimics a tenuous viral infection, promoting a viral immune response and a minimal bacterial immune response. This mechanism could predispose the bovine to secondary bacterial infections during the establishment of vaccine immunity, which occurs 1-3 weeks after the last dose of vaccination (Gomes et al. 2015).

Parenteral vaccines stimulate the local immune response to a lesser extent than IN vaccines (Neutra and Kozlowski 2006). Compared to the IN route, the challenge to the nasal mucosa is lesser when the intramuscular (IM) route is used. Hence, it is believed that the IM route results in less susceptibility to secondary bacterial infections during the vaccine challenge period.

There have been many studies on bovine vaccines against respiratory viruses comparing different formulations, doses, and routes. These studies mainly focused on antibody production and seroneutralization after the establishment of vaccine immunity, and almost none of them focused on the effects of the vaccine on bronchoalveolar cells and the possible immune susceptibility to bacterial diseases during establishment of the vaccine response (Gershwin et al. 1998; Fulton et al. 2003; Hishiki et al. 2004).

Thus, the present study aimed to compare the effects of two commercial vaccine protocols, defined by the manufacturers’ recommendations, on the bronchoalveolar fluid of healthy heifers in the pasture system.

## 2. Material and methods

This experiment was approved by the UNICENTRO Animal Ethics Committee (018/2017). The study was conducted at a dairy farm of UNICENTRO (Universidade Estadual do Centro Oeste), located in Guarapuava, Paraná, Brazil.

### 2.1. Animals and feeding

21 healthy Jersey heifers aged 15 ± 2 (SD) months and weighing 200 ± 50 kg, without previous vaccination and with negative serology for BVDV virus before the experiment (ELISA test BVDV total ab test and ELISA test BVDV antigen Idexx, São Paulo, SP, Brazil) were used. The animals were dewormed 30 days before the experiment (Ivomec, 1 ml/50 kg, Merial, São Paulo, SP, Brazil). The animals were fed a total mixed ration of 0.8 corn silage and 0.2 concentrate (Leitemax 18, CooperativaAgrária, Guarapuava, PR, Brazil), and mineral salt (Bovigold, Tortuga, Sao Paulo, SP, Brazil) on a dry matter (DM) basis twice a day (Table 1). In the intervals between feedings, the cows remained on pasture of ryegrass (*Lolium multiflorum)*.

**Table 1.** Chemical composition of the corn silage used in animal feed.

Parameters	Corn Silage	Concentrate	Experimental diet
Dry matter content(% of natural matter)	37.17	90	47.36
Crude protein (% of DM)	7.9	42.2	14.7
Mineral matter (% of DM)	3.95	8	4,76
Crud protein brut (% of DM)	7.79	18	25.41
Neutral detergent fiber (% of DM)	55.62	11.0	46.72
Acid detergent (% of DM)	30.02	0	24.01
Either extract(% of DM)	3.47	2.5	3.27
Total digestible nutrients (% of DM)	66.86	4.73	54.34

Diet composition: 80% of corn silage and 20% concentrate. Mineral and vitamin mixture: Ca = 190 g/kg; *p* = 60 g/kg; Mg = 20 g/kg; Cl = 167 g/d; K = 335 g/kg; Na = 70 g/kg; S = 20 g/kg; Co = 15 mg/kg; Cu = 700 mg/kg; Fe = 700 mg/kg; Mn = 1600 mg/kg; Se = 19 mg/kg; Zn = 2500 mg/kg; Cr = 10 mg/kg; Vit.A = 400,000 IU/kg; Vit.D = 100,000 IU/kg; Vit.E = 2400 IU/kg.

### 2.2. Experimental design

Only healthy animals were included in the experiment based on the findings of blood count, physical examination, and bronchoscopic evaluation. These animals were randomly distributed in equal numbers in the control (CO), IN, and IM groups, according to the adopted vaccination protocol. The CO did not receive the vaccine and was not manipulated until the first evaluation. The IN group received 1 mL of the commercial vaccine (Inforce 3®, Zoetis, SP São Paulo, SP, Brazil) in each nostril, in a single dose. For this purpose, the animals were contained in a containment trunk, and their heads were held for one min for administration and absorption of the vaccine. The vaccine composition was BoHV-1, bPIV-3, and BRSV (attenuated samples). The IM group was vaccinated with two doses of 2 mL with an interval of 21 days of the commercial vaccine, composed of viruses that contained attenuated BoHV-1, bPIV-3, BRSV (attenuated sample), and BVDV (inactivated sample) viruses, composition of 5960 cytopathic and 6309 non-cytopathic strains (Cattle Master 4®, Zoetis, São Paulo, SP, Brazil).

### 2.3. Sample analysis

The animals were subjected to bronchoalveolar evaluations by inspection of the respiratory tract and collection of bronchoalveolar lavage fluid (BALF) for analysis of cytological profile, measurement of oxidative metabolism, and quantification of IgA and IgG, at day 0 (D0), just prior to the application of the first vaccine dose, and on D3, D7, and D21, after either the IN vaccine dose or the second IM vaccine dose.

### 2.4. Evaluation of respiratory tract irritation

**Figure 1.**
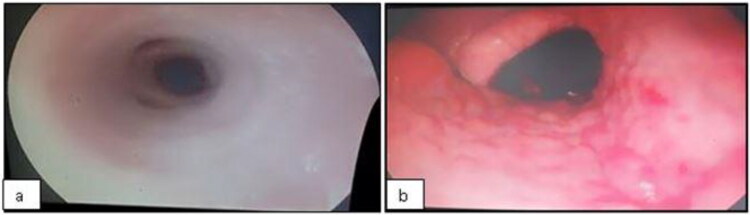
The respiratory tract irritation score: score 1 - smooth and shiny pink mucous membranes without secretions in nasopharynx or trachea (a); score 2 - reddish mucosa, evidenced vessels, and irregularities in nasopharynx (b).

For bronchoscopy, the animals were sedated with xylazine (Anasedan, cevaBRA, São Paulo, SP, Brazil) at a dose of 0.02 mg/kg BW, intravenously. Endoscopic examination and BALF samples were then performed using a flexible videoendoscope (EC-250LP5, Fujinon1, 690 mm × ø11 mm, Miami, FLO, United States), passed through one of the nostrils until it reached the region of the bronchi, and continued until resistance was detected. The respiratory tract irritation was evaluated as follows: score 1 – smooth and shiny pink mucous membranes without secretions in the nasopharynx or trachea (Figure 1a); score 2 – reddish mucosa, evidence of vessels, and irregularities in nasopharynx (Figure 1b); score 3 – reddish mucosa, evidence of vessels, and irregularities in trachea; and score 4 – reddish mucosa, evidence of vessels, and irregularities in nasopharynx and trachea.

### 2.5. Bronchoalveolar lavage sample

After inspection of the respiratory tract, BALF was promoted by administering 30 mL of sterile 0.9% saline at room temperature in each bronchus. The solution was suctioned by a vacuum pump, recovering approximately 40 mL of BALF, according to the technique described by Batista et al. (2012). Subsequently, the samples were stored in polyethylene tubes and cooled, and processed within 2 h after collection. The BALF samples were centrifuged at 1000 × g for 15 min at 4 °C, and were supernatant conditioned and frozen for measurement of IgA and IgG by a commercial ELISA kit (190516 IgA Cow ELISA Kit and 190517 IgG Cow ELISA Kit, Abcam, Cambridge, MA, United States), as recommended by the manufacturer, at dilutions of 1:2 and without dilution, respectively. The cell pellet was then suspended in 1 mL of phosphate buffered saline (PBS) at 4 °C and subjected to cell viability and counting, using the leukocyte count technique. All samples showed viability greater than 90%, and the cell suspension was adjusted to 2 × 10^6^ viable cells/mL for the evaluation of cell metabolism and cytology.

### 2.6. Oxidative metabolism

To assess oxidative metabolism, the colorimetric nitroblue-tetrazolium test (NBT) was performed (Artner et al. 2018). We added 100 µl of BALF (2 × 10^6^ viable cells/mL) to 100 µL of 1% NBT (Sigma, São Paulo, SP, Brazil), stimulated with 5 µL of 12-myristate 13-phorbol myristate acetate (PMA 300 ng/mL, Sigma) in test tubes, and incubated for 30 min at 37 °C. After stopping the reaction by adding 2000 µL of cold EDTA (3 M), the cells were suspended in PBS 1000 µL, and the external NBT was removed after washing 1000 µL, with methanol (Synth São Paulo, SP, Brazil). The cells were dissolved with KOH (Synth) (3 M, 120 µL) and DMSO (Dimesol, Marcolab São Paulo, SP, Brazil) (99%, 140 µL), and the microplates were read using an ELISA reader (Thermo Plate TP – Reader São Paulo, SP, Brazil) with a 630 nm filter.

### 2.7. Cytological evaluation

For cytological evaluation, 200 µL of BALF was cyto-centrifuged at 400 x g for six min. Pellet cells were fixed with methanol (Synth, São Paulo, SP, Brazil) and stained with Panoptic Rapid (São Paulo, SP, Brazil). For differential cell counting, an optical microscope was used. 300 cells were counted using 1000x magnification and were classified into simple macrophages, giant macrophages, neutrophils, lymphocytes, epithelial cells, and eosinophils.

### 2.8. Statistical analysis

The data collected for each variable were subjected to analysis of variance (ANOVA) using the statistical software Instat Graphpad, (GraphPad Software, La Jolla, CA, USA). For the evaluation of the differences between the means, parametric repeated measurement ANOVA test and the Tukey test were performed for time interaction, and ANOVA ordinary test and Tukey test were performed for treatment interaction. The nasotracheal irritation scores were transformed into the animal frequency of each score. This data was analyzed using the Chi-square test. The data were considered statistically significant when *p* < 0.05.

## 3. Results

Before the start of the experiment, two heifers from the IM and two from the CO group developed leukocytosis and neutrophilia without respiratory alterations at day 0 and were thus removed from the experiment. Thus, the experiment was carried out with five animals in the CO group, seven in the IN group, and five in the IM group. All animals included in the experiment displayed no alterations in clinical exam or hematological parameters. At D0, the animals showed no changes in the clinical examination of the respiratory tract or in bronchoalveolar cytology.

**Figure 2.**
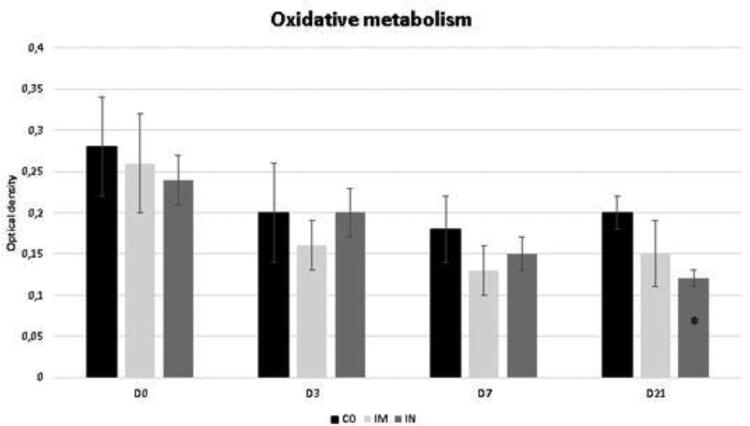
Oxidative metabolism in cells collected from BALF of healthy Heifers vaccinated against the bovine respiratory complex (BRD) compared to controls. Means, Error bars present SEM from the mean Statistical significance was assessed by Tukey test. * *p* < 0.05, treatment interaction. Time interaction: CO p = 0.11; IM p = 0.26; IN p = 0.07). CO (group control; n = 5); IM (group vaccinated against BRD intramuscularly; n = 5); IN (group vaccinated against BRD intranasally; n = 7). (D0) is day 0 immediately before the application of the first dose of the vaccine; (D3, D7 and D21) are three, seven and 21 days respectively after the last dose of the vaccine.

The data regarding the oxidative metabolism of bronchoalveolar cells is shown in Figure 2. This variable did not show a significant difference for time interaction. The IN group showed a lower oxidative metabolism of bronchoalveolar cells at D21 compared to the CO group (*p* = 0.04).

**Figure 3.**
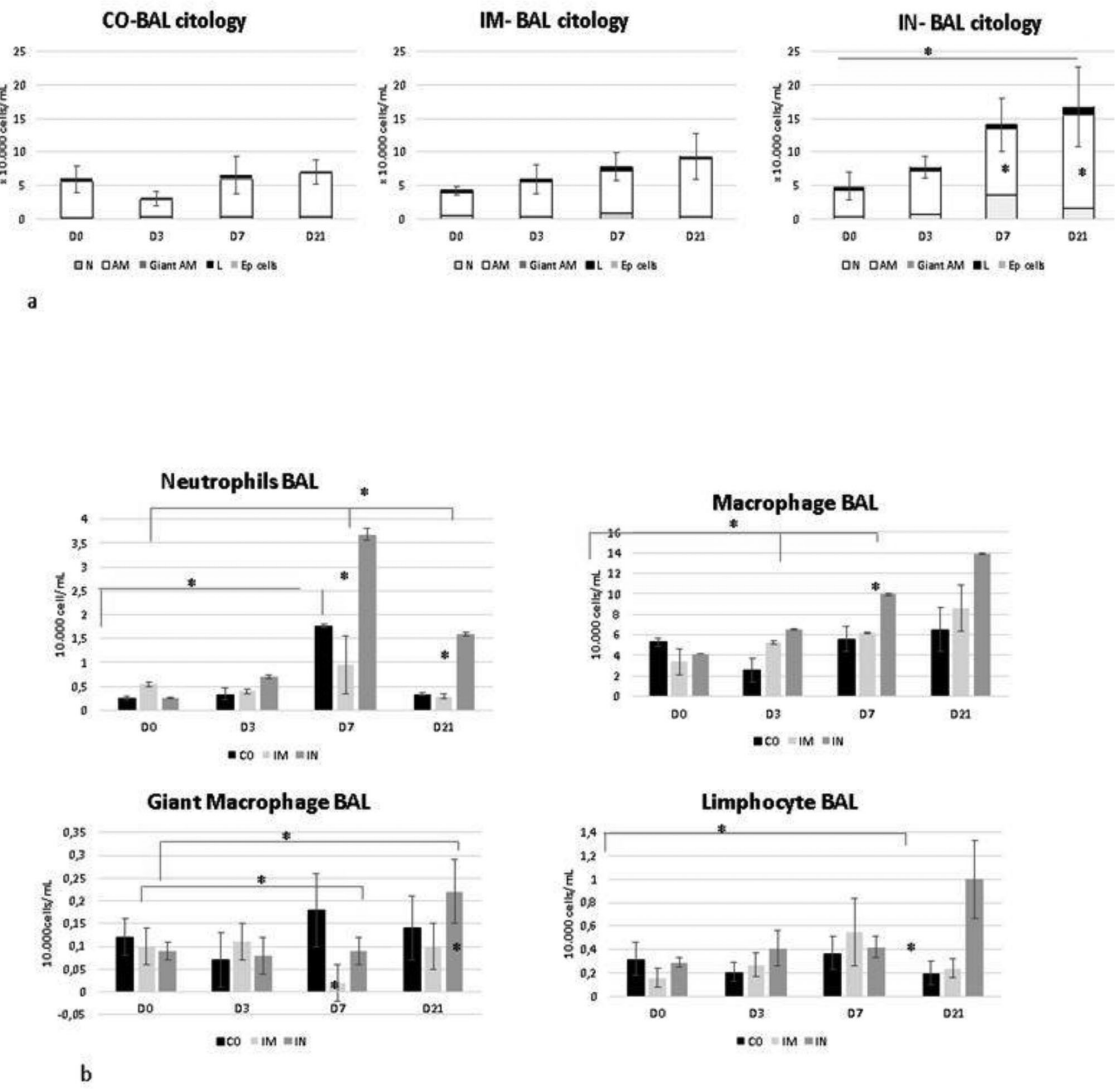
Cells collected from BALF of healthy Heifers vaccinated against the bovine respiratory complex (BRD) compared to controls. Data from healthy Heifers submitted to vaccines against the bovine respiratory complex (BRD). (a) Total values of bronchoalveolar cells -Means, Error bars present SEM of total cellularity. (b) Total values of individual bronchoalveolar cells with Statistical significance. N = neutrophils, AM- alveolar macrophage, Giant am- giant alveolar macrophage, L- limphocytes, Ep cells- epetelial cells. CO (control group; n = 5); IM (group vaccinated against BRD intramuscularly; n = 5); IN (group vaccinated against BRD intranasally; n = 7). (D0) is day 0 immediately before the application of the first dose of the vaccine; (D3, D7 and D21) are three, seven and 21 days respectively after the last dose of the vaccine. Means statistical significance was assessed by Tukey test. * *p* < 0.05, inner the graphics bars- treatment interaction, outside the graphics bars- time interaction.

Bronchoalveolar fluid cytology is shown in Figure 3. At D0, BALF cytology was predominantly composed of alveolar macrophages, (84% simple macrophages, and 2% giant alveolar macrophages), followed by neutrophils (7.5%), lymphocytes (5.3%), eosinophils (1.7%), and epithelial cells (0.6%), in all groups.

There was a significant (*p* = 0.03) increase in cellularity in the IN group at D7 and D21 compared to the other groups and to other time points (D0 and D3). This higher cellularity was caused by an increase in neutrophils at D7 and by neutrophils, alveolar macrophages, and lymphocytes at D21 (neutrophil with *p* = 0.03 treatment interaction and *p* = 0.02 time interaction; alveolar macrophage with *p* = 0.02 time interaction and *p* = 0.04 treatment interaction; giant alveolar macrophage with *p* = 0.01 time interaction and without treatment interaction; and lymphocytes with *p* = 0 treatment interaction and *p* = 0.04 time interaction).

IM vaccination showed no change in cellularity over time points, however, there was a reduction of giant alveolar macrophages at D7 compared to D0 and D3 (*p* = 0.04) in the time interaction and in comparison to the other groups (*p* = 0.02). The CO group also showed no increase in cellularity, although there was a significant increase in neutrophils at D7 compared to other time points (*p* = 0.04).

**Figure 4.**
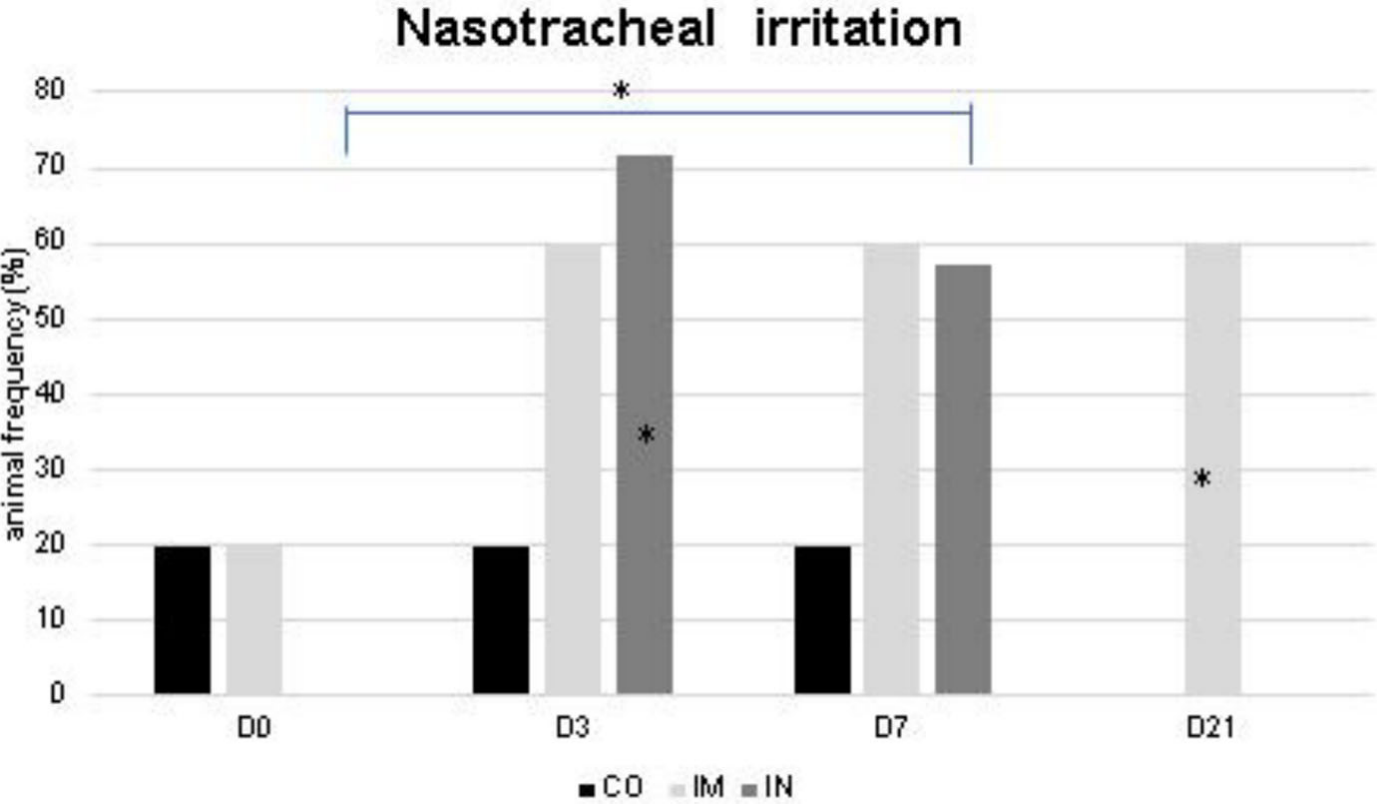
Absence of irritation in the nasotracheal mucosa of healthy Heifers vaccinated against the bovine respiratory complex (BRD) compared to controls. CO (group control; n = 5); IM (group vaccinated against BRD intramuscularly; n = 5); IN (group vaccinated against BRD intranasally; n = 7). (D0) is day 0 immediately before the application of the first dose of the vaccine; (D3, D7 and D21) are three, seven and 21 days respectively after the last dose of the vaccine. Statistical significance of frequency was assessed by Chi square test. * *p* < 0.05, inner the graphics bars- treatment interaction, outside the graphics bars- time interaction.

The data from the frequency of irritation in the nasotracheal region is shown in Figure 4. More animals with nasopharyngeal irritation in the IN group at D3 and D7 were observed compared to other time points (*p* = 0.03), and compared to the CO group at D3 (*p* = 0.03). In the IM group, there was an increase in animals with nasopharyngeal irritation at D21 compared to the CO group (*p* = 0.006).

**Figure 5.**
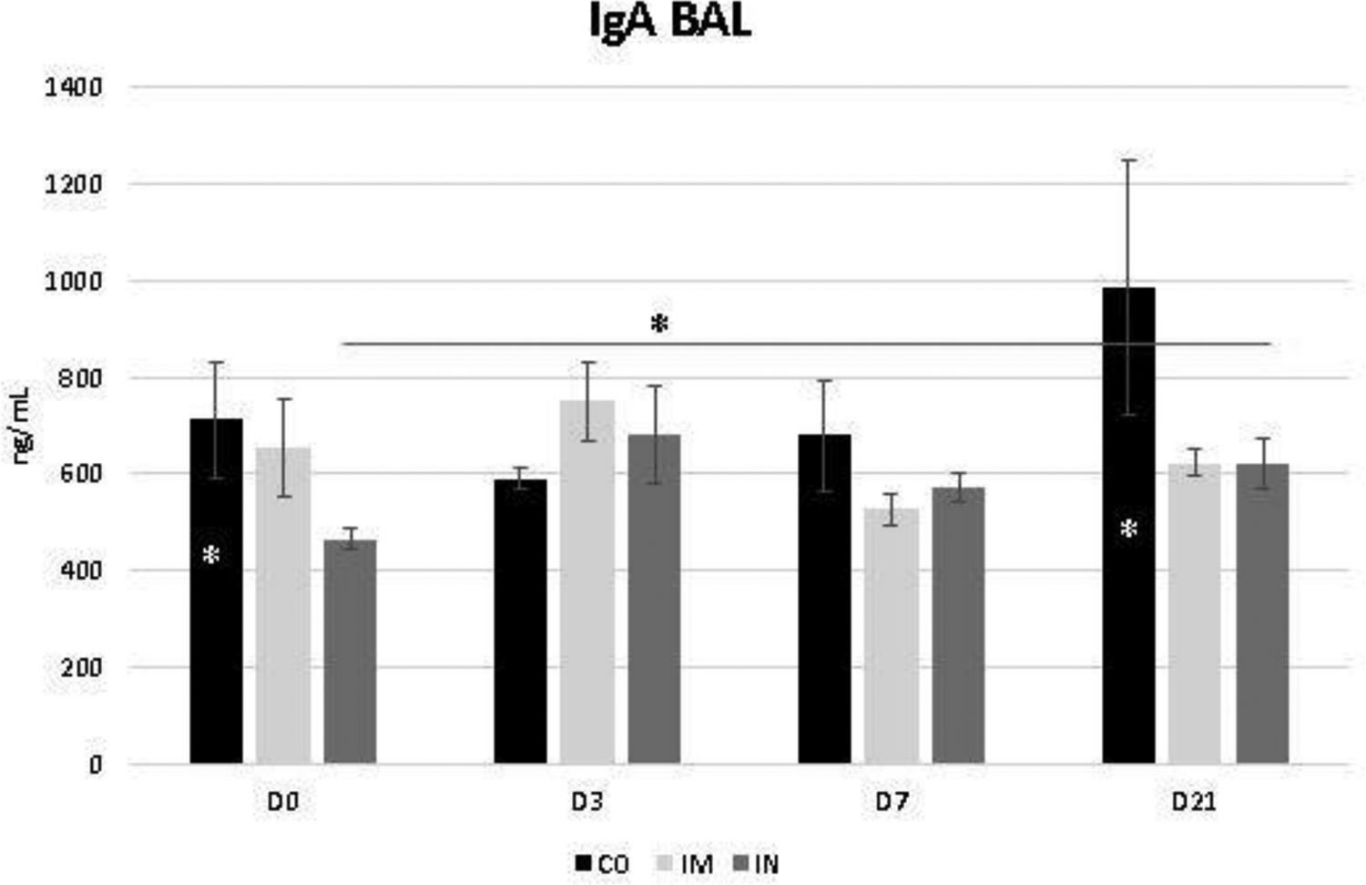
Presence of IgA in bronchoalveolar lavage fluid (BALF) in healthy Heifers vaccinated against the bovine respiratory complex (BRD) compared to controls. Means statistical significance was assessed by Tukey test. * *p* < 0.05, inner the graphics bars- treatment interaction, outside the graphics bars- time interaction. CO (group control); IM (group vaccinated against BRD intramuscularly); IN(group vaccinated against BRD intranasaly). (D0) is day 0 immediately before the application of the first dose of the vaccine; (D3, D7 and D21) are three, seven and 21 days respectively after the last dose of the vaccine.

**Figure 6.**
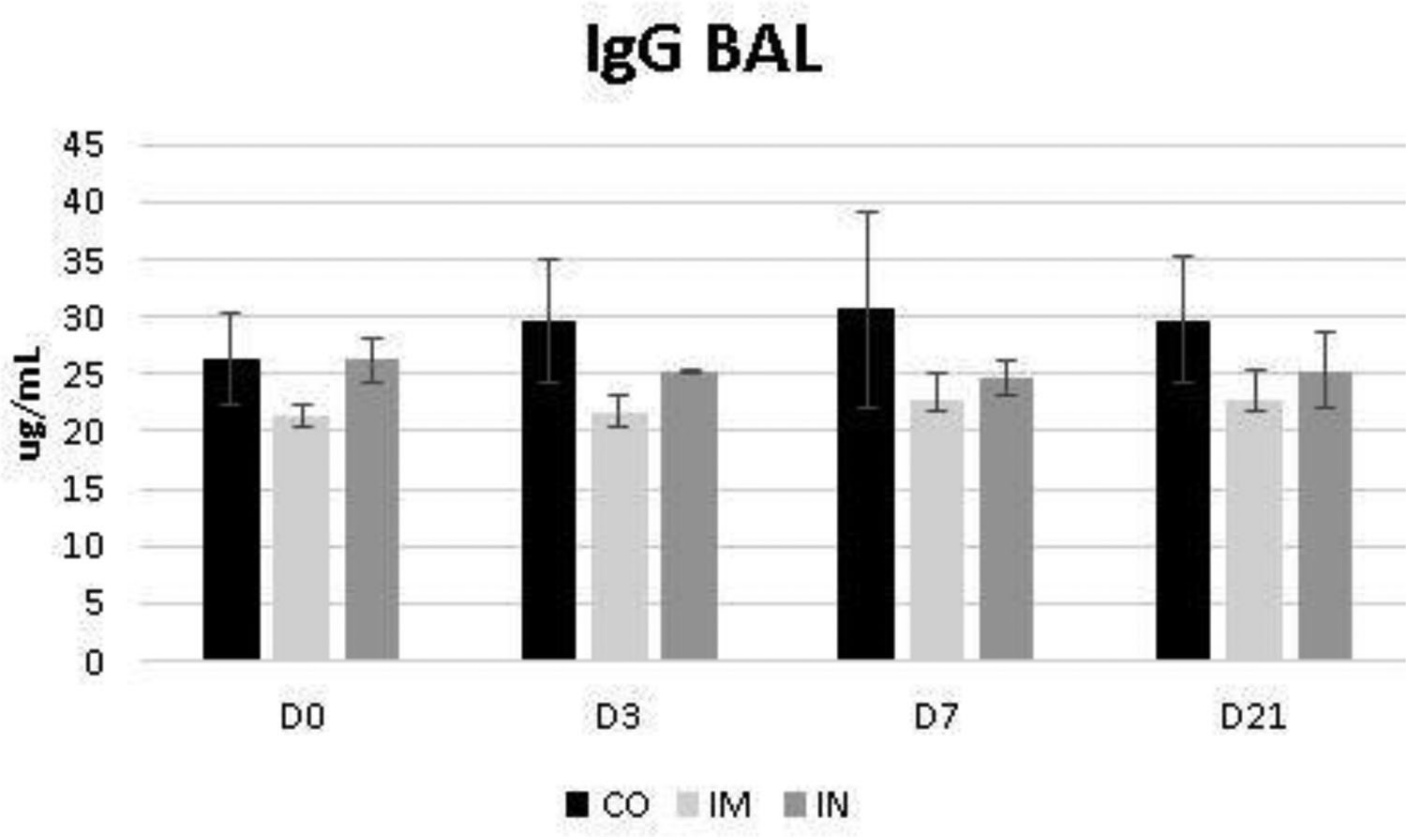
Presence of IgG in bronchoalveolar lavage fluid (BALF) in healthy Heifers vaccinated against the bovine respiratory complex (BRD) compared to controls. CO (group control; n = 5); IM (group vaccinated against BRD intramuscularly; n = 5); IN (group vaccinated against BRD intranasally; n = 7). (D0) is day 0 immediately before the application of the first dose of the vaccine; (D3, D7 and D21) are three, seven and 21 days respectively after the last dose of the vaccine. Means statistical significance was assessed by Tukey test. * *p* < 0.05, inner the graphics bars- treatment interaction, outside the graphics bars- time interaction.

The data regarding the bronchoalveolar contents of IgA and IgG are shown in Figure 5 and Figure 6, respectively. While the levels of BALF IgG were not influenced by the vaccination protocol, it was noted that the levels of BALF IgA increased significantly at D3 in the IN group (*p* = 0.04) for time interaction. Despite this, the IgA level of the IN group did not exceed the CO group levels at most of the evaluated times, whose levels were already significantly higher at D0 compared to the other groups (*p* = 0.03) (Figure 5). These levels in the CO group remained stable at D3 and D7 and increased by D21 compared with the other groups (*p* = 0.03).

## 4. Discussion

The IN vaccination protocol against BRD caused more changes in the respiratory tract than the IM vaccination protocol during the establishment of vaccine immunity. The IN protocol increased bronchoalveolar IgA levels and promoted irritation of the nasopharynx up to three days after the vaccine. There was an increase in cellularity with changes in the cytological profile, and reduced oxidative metabolism until D21, probably because the IN vaccination protocol caused an exposure of the respiratory tract to pathogenic agents, which may have caused the alteration in the cellular and humoral profile to protect the respiratory tract against the action of these pathogenic microorganisms (Fulton et al. 2003; Socha et al. 2013; Gomes et al. 2015; Walz et al. 2017).

As the vaccine formulations used in this experiment contain modified live viral agents such as BoHV-1, bPVI-3, and BRSV, it is possible that attenuation methods might reduce the pathogenicity of the virus, but still maintain their ability to colonize and multiply in the host tissues. This can cause impairment of some immune functions, and consequently, can promote disease in the host (Schatzmayr 2003, Meeusen et al. 2007).

Oxidative metabolism is an oxygen-dependent antibacterial mechanism, which also occurs in neutrophils, eosinophils, and monocytes (Fonteque et al. 2015). This function is initiated by cell phagocytosis, followed by the production of reactive oxygen species (ROS), which promote the elimination of internalized pathogens (Burgos et al. 2011; Fonteque et al. 2015). This mechanism is important to keep the respiratory tract healthy, and if this activity is reduced, it is difficult to eliminate infectious agents, consequently increasing the chance of developing bacterial pneumonia (Du Manoir et al. 2002; Bertagnon et al. 2017). Thus, the results of the present study demonstrate that the IN vaccination protocol led to a reduction in oxidative capacity of bronchoalveolar cells at the pulmonary level, leaving pathogens inhaled alive in the posterior respiratory tract and making it more prone to infections during the establishment of vaccine immunity.

Additionally, during the same time period, there was a significant increase in cellularity in the IN group, with an increase in neutrophil count. This influx may have occurred because neutrophils are the first defense cells to be recruited during an inflammatory state, to protect the respiratory tract in response to stimuli caused by microorganisms or inhaled particles in the respiratory system (Griffin et al. 2010).

This fact has already been reported by Gomes (2016), who used a commercial vaccine against BRD via the IN route in newborn calves, suggesting that this route of administration reduces the defenses of the respiratory tract, which induces a compensatory neutrophilic influx with increased regional cellularity to keep the respiratory tract healthy. In addition, during this period, the IN group presented nasopharyngeal irritation, which reinforces the hypothesis that the IN vaccine protocol promotes irritation and inflammation in the respiratory tract.

Although the CO group also showed a significant increase in neutrophils and a decrease in simple macrophages at D7, there was no change in other variables such as alveolar macrophages, cellularity, irritation of the nasopharynx, or oxidative metabolism, indicating that the repeatability of bronchoscopy causes mild inflammation in the region, either by carrying microorganisms from the anterior respiratory tract to the posterior region or by local abrasion caused by the passage of the endoscope. This irritation, which is mechanical or biological, leads to the release of chemotactic substances that mobilize inflammatory cells to the injured site, mainly neutrophils, proportional to the degree of inflammation generated. By D21, there was a return to normal proportions between macrophages and neutrophils, and it is believed that the 15-day period between D7 and D21 was enough time to resolve the inflammation generated by the technique (Vianna et al. 2016; Shecaira 2017).

Regarding the humoral immune response, it is emphasized that IgA is the main immunoglobulin produced in mucous membranes. Its main function is to trap antigens or microorganisms in the mucus, preventing direct contact of pathogens with the mucosal surface. Although IgA does not have a bactericidal action, it neutralizes viruses and viral and bacterial enzymes, in addition to preventing bacteria from penetrating mucous membranes (Mora et al. 2006; Tizard 2014).

In view of this, the increase in bronchoalveolar IgA levels during the initial days in the IN group is in agreement with a previous study by Cortese et al. (2017), who observed that cows vaccinated with a modified live intranasal vaccine, containing BoHV-1, BRSV, and bPIV-3, showed a significant increase in levels of IgA in their nasal secretions during the initial days after vaccination (4 and 7 days after vaccination).

IgA secretion can also be stimulated by the BALF collection technique, since the endoscope carries a considerable number of microorganisms to the lower respiratory tract, leading to local production of immunoglobulins or causing lesions in the subepithelial wall of the respiratory tract with extravasation of serum constituents for the pulmonary lumen (Bertagnon et al. 2007). Thus, it is believed that the continued use of the technique stimulated the production of immunoglobulin in the CO group. However, there was no reduction in the cellular immune response or consumption of this immunoglobulin. In the IN group, however, the absence of an increase in this immunoglobulin at D7 and D21, and the increase in lymphocytes at D21, could indicate a consumption of IgA locally for pathogen neutralization despite the lower efficiency of the cellular immune response.

Although the immune change in BALF in the IN group was higher in comparison to other groups, no animal had clinical manifestations of respiratory disease or alteration in the blood count. Thus, it is inferred that as the handling conditions of the animals in this study presented a low environmental challenge, the IN vaccination protocol did not increase the occurrence of secondary bacterial infections. This is similar to the results of Xue et al. (2010), where they used vaccines with live modified viruses intranasally for calves, against respiratory viruses. In the present study, there was a minimal stressful condition, with the heifers, with a mature immune system, stocked in pastures, and in good nutritional conditions; this situation differed from the situation of the feedlot cattle or newborn calves, where the population was more affected by BRD (Edwards 2010).

Thus, it is suggested that studies also be carried out on cattle exposed to a greater environmental challenge because there are reports that vaccines against respiratory viruses applied via the IN route generate viral shedding in nasal secretions after vaccination in feedlot cattle (Socha et al. 2013) and these vaccines have been associated with an increased incidence of pneumonia in dairy calves (Ollivett et al. 2018).

In the IM group, the only changes found were a reduction in giant alveolar macrophages at D7 and increased irritation in the nasopharynx from D3 until D21. Furthermore, the IM vaccine protocol did not promote statistical changes in oxidative metabolism or in the production of IgA and IgG in the bronchoalveolar region. This indicates that the IM vaccine protocol also promoted changes, to a lesser magnitude than the IN protocol, in the respiratory tract. Previous studies have found that mucosal immunization pathways induce a greater production of IgA at the application site, although the effects of the vaccine on bronchoalveolar cell defense have not been studied. (Neutra and Kozlowski 2006; Ellis et al. 2007; Cortese et al. 2017).

Similarly, Fulton et al. (2003) have shown that animals vaccinated via IM with a modified vaccine against BoHV-1 and BVDV-1, displayed increased blood leukocytes 3-7 days after the second vaccine dose, although they did not transmit viruses to the non-vaccinated animals that remained together.

In the present experiment, no animal had a positive BVDV titer at D0, and only the IM group showed BVDV titers at D21. While both vaccines contained the same attenuating viral agents in their formulations, only the IM protocol had inactivated BVDV. It is believed that in herds where BVDV is circulating, the undesirable effects of the IN vaccine protocol may be more important, since this reduction in cell activity, without the appropriate humoral protection against the disease, would favor a greater occurrence of BVDV.

Although reports on the ability of vaccines against bovine respiratory diseases administered by IN or parenteral route are still scarce, concerns remain regarding the possibility that the attenuated viruses can reverse virulence (Schatzmayr 2003; Meeusen et al. 2007; Socha et al. 2013), making it necessary to carry out further studies with different environmental challenges and the use of commercial vaccines against respiratory viruses.

## 5. Conclusion

We concluded that the IN and IM vaccine protocols against the BRD used in the present study, produced bronchoalveolar inflammation during the establishment of the vaccine immunity, which was more expressive in the IN protocol.
